# Feedback Loop of DUXAP8/miR-214-3p/KLF13 Facilitates Hepatocellular Carcinoma Progression and Serves as an Indicator of Tumor Microenvironment via Impacting Piezo1

**DOI:** 10.3390/ijms27114873

**Published:** 2026-05-28

**Authors:** Yifan Zhang, Xinyi Luo, Yiquan Lu, Fengjie Hao, Xiaochun Fei, Yongjun Chen, Junqing Wang

**Affiliations:** 1 Department of General Surgery, Ruijin Hospital, Shanghai Jiao Tong University School of Medicine, 197, Rui Jin Er Road, Shanghai 200025, China; graduated_fan@163.com (Y.Z.); teamlabmate_2@126.com (X.L.); teamlabmate_5@126.com (Y.L.); teamlabmate_4@126.com (F.H.); 2 Department of Pathology, Ruijin Hospital, Shanghai Jiao Tong University School of Medicine, 197, Rui Jin Er Road, Shanghai 200025, China; teamlabmate_1@126.com

**Keywords:** pseudogene *DUXAP8*, feedback loop, tumorigenesis, HCC, *Piezo1*

## Abstract

Pseudogenes are barely transcribed normally, but some of them are transcribed as long non-coding RNAs during tumorigenesis. The pathological features of pseudogenes in hepatocellular carcinoma (HCC) have not been illustrated clearly. Here, we engaged the pseudogene *DUXAP8* into a feedback loop affecting the HCC indicator *Piezo1* and concerning the HCC tumor microenvironment (TME). As we discovered, the *DUXAP8* transcript was detectable in HCC. The transcriptional activity of *DUXAP8* in HCC is associated with dismal HCC clinicopathological features. By depleting *DUXAP8*, the HCC cells presented the inhibition of cell proliferation along with significant cell apoptosis in vitro and potently suppressed the HCC tumorigenesis ability in vivo. Combined with the ChIP assay, the direct interaction of either the *DUXAP8* transcript/*miR-214-3p* or *miR-214-3p*/*KLF13* mRNA was verified, and the promoting effect of KLF13 on *DUXAP8* transcription was also validated, which further illustrates a positive feedback loop of *DUXAP8*/*miR-214-3p*/*KLF13*. Moreover, KLF13 was found to facilitate *Piezo1* transcription. As concluded, our findings suggest that the pseudogene *DUXAP8* promotes HCC tumorigenesis through the feedback loop of *DUXAP8*/*miR-214-3p*/*KLF13* and participates in HCC TME modulation by impacting *Piezo1*.

## 1. Introduction

Hepatocellular carcinoma (HCC) engages in tumor development, invasion, and metastasis, which are difficult to control [1]. Poor outcomes and high mortality occur in HCC patients due to the potent tumor heterogeneity and aggressive cell biological behavior [2]. Recently, based on HCC treatment developments, including targeted therapy and tumor immunotherapy, definitive results in HCC control have been achieved, especially for those patients in the later stages, via the introduction of targeted therapy and tumor immunotherapy. However, the overall survival (OS) of HCC patients is far from satisfactory [3]. The discovery of innovative and practical targets for HCC prevention and treatment is imperative.

The tumor microenvironment (TME) of HCC is a complex internal condition with hallmarks of irregular angiogenesis, uncontrolled chronic inflammation, and the abnormal remodeling of the extracellular matrix (ECM) [4]. The indispensable pathophysiological background provided by TME spans from cancer initiation to distant metastasis. And the elusive states of TME contribute to the diverse mechanisms of drug resistance, immune escape, and treatment failure by interacting with tumor cells [5,6]. Notably, TME involves diverse oncogenic signaling pathways in the development of tumors. As such, it can promote intercellular communication by releasing paracrine signals from cytokines, chemokines, growth factors, and proteases. This suggests that TME holds promise for discovering innovative targets and therapeutic means by elucidating potential molecular mechanisms.

The competing endogenous RNA (ceRNA) effect is an indirect regulatory mechanism of gene expression, where competing RNA molecules bind to the same microRNA (miRNA) and dampen the suppression effect. Currently, accumulating evidence has found that ceRNA affects various TMEs. Our studies have demonstrated that HCC is particularly involved with this pseudogene-related regulation [7]. Recently, Ren et.al. reported that Piezo-type mechanosensitive ion channel component 1 (Piezo1), a broadly expressed membrane protein that triggers biological signals, induced by mechanically activated ion channels, was upregulated in HCC and plays a pivotal role in a feedback loop with integrin β1, HIF-1α, and VEGF, which indicates a poor prognosis for HCC patients, concerning the matrix-stiffness-related TME. However, a comprehensive understanding of how *Piezo1* regulates the HCC TME is still lacking.

In this study, we noticed the irregular transcription of the pseudogene *DUXAP8* and elucidated a feedback loop interplaying with *microRNA-214-3p* (*miR-214-3p*, a suppressor of HCC) and KLF13 (KLF Transcription Factor 13, a transcription factor promoting *Piezo1* expression in HCC). To our discovery, *DUXAP8* is a tumor-promoter, undetectable in non-cancerous liver tissues, while extremely ascending in HCC. Based on the suppressive effects on tumor growth by knocking down *DUXAP8* in HCC cells, we suggest that the feedback loop of *DUXAP8*/*miR-214-3p*/*KLF13* is a potential indicator of HCC TME through enhancing *Piezol* transcription. The members of the feedback loop could be promising targets for HCC treatment.

## 2. Results

### 2.1. DUXAP8 Transcript Was Potently Upregulated in HCC Cell Lines and Tissues

**Figure 1:**
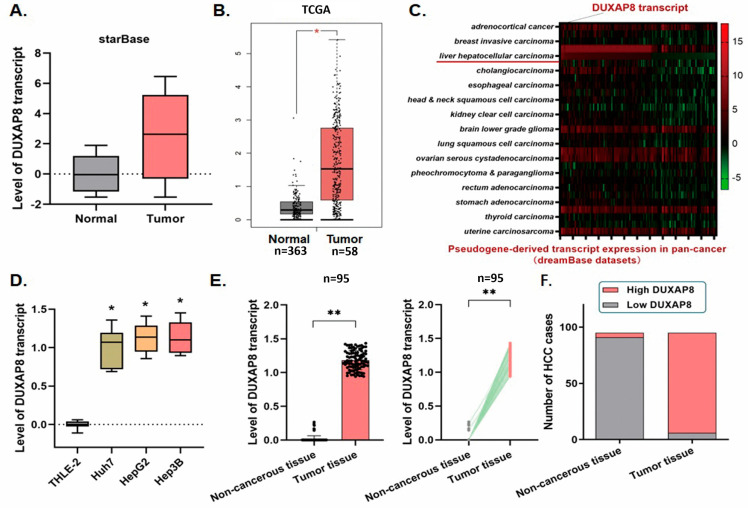
The pseudogene transcript level of *DUXAP8* in HCC. (**A**) The transcript of the pseudogene *DUXAP8* in HCC was detected by analyzing the relevant HCC dataset from the starBase database. A high level of *DUXAP8* transcript in the HCC tissues was illustrated (*p* < 0.001). (**B**) The *DUXAP8* transcript in HCC was detected by analyzing the TCGA database (*n* = 421, * *p* < 0.05). Transcription of *DUXAP8* was activated and highly expressed in the HCC tissues (*p* < 0.0001). (**C**) The signature genes in HCC with expression changes were detected by analyzing the dreamBase dataset (expressions in HCC outlined in red), and the heatmap was generated. The *DUXAP8* transcript was detected at a higher level in HCC tissues than in normal tissues. (**D**) RT-qPCR assay demonstrated that *DUXAP8* was activated and significantly highly expressed in HCC cell lines in comparison with the control THLE-2 cells (* *p* < 0.05). (**E**) RT-qPCR assay was conducted on the 95 real patients’ specimens. The *DUXAP8* transcript was highly expressed in the tumor tissues, and only a few non-cancerous tissues presented detectable *DUXAP8* transcript at a lower level (** *p* < 0.01). (**F**) Statistic of the number of cases concerning the *DUXAP8* transcription in HCC specimens. A significant increase in *DUXAP8* transcript was detected in most HCC tissues (89/95, 93.68%), whereas, in non-cancerous tissues, only a small proportion (4/95, 4.21%) showed a low level of detectable *DUXAP8* transcript.

The analysis of the dreamBase, LCLE databases, and TCGA liver cancer datasets shows that the *DUXAP8* transcript is detectable at a high level in the HCC tumor tissues, but is barely transcribed in normal liver tissues (Figure 1A–C). In the recruited HCC cell lines (Huh7, HepG2, and Hep3B), the *DUXAP8* transcript was detected at a relatively high level in general, compared with the control THLE-2 cells, which showed an almost undetectable expression of *DUXAP8* (Figure 1D,E).

The detection of the real patients’ specimens collected from our medical center demonstrated that *DUXAP8* was transcribed in all the HCC tissues, with a portion of 93.68% (89/95) specimens that presented a significantly high *DUXAP8* transcript level, and only a small portion (6.32%, 6/95) of a relatively lower level. In contrast, the *DUXAP8* transcript was detectable in only 4.21% (4/95) of the adjacent non-cancerous liver tissues at an extremely low level (Figure 1F).

### 2.2. DUXAP8 Transcript Is Correlated with the Dismal Clinicopathologic Features in HCC Patients

The correlation between *DUXAP8* transcription and the clinicopathologic features of the 95 HCC patients was statistically analyzed. Table 1 demonstrates that no significant correlation between the *DUXAP8* transcript and the patient’s age, gender, tumor size, and virus control status was observed, while the highly transcribed *DUXAP8* shows a positive relationship with the serum Alpha-fetoprotein (AFP) levels (*p* < 0.05), more advanced TNM stages (*p* < 0.05), tumor microsatellite formation (*p* < 0.05), and venous invasion (*p* < 0.05). These findings indicate that the *DUXAP8* transcript plays a promoting role in HCC.

### 2.3. Knockdown of DUXAP8 Transcript Impairs HCC Cell Proliferation and Induces Cell Apoptosis

**Figure 2:**
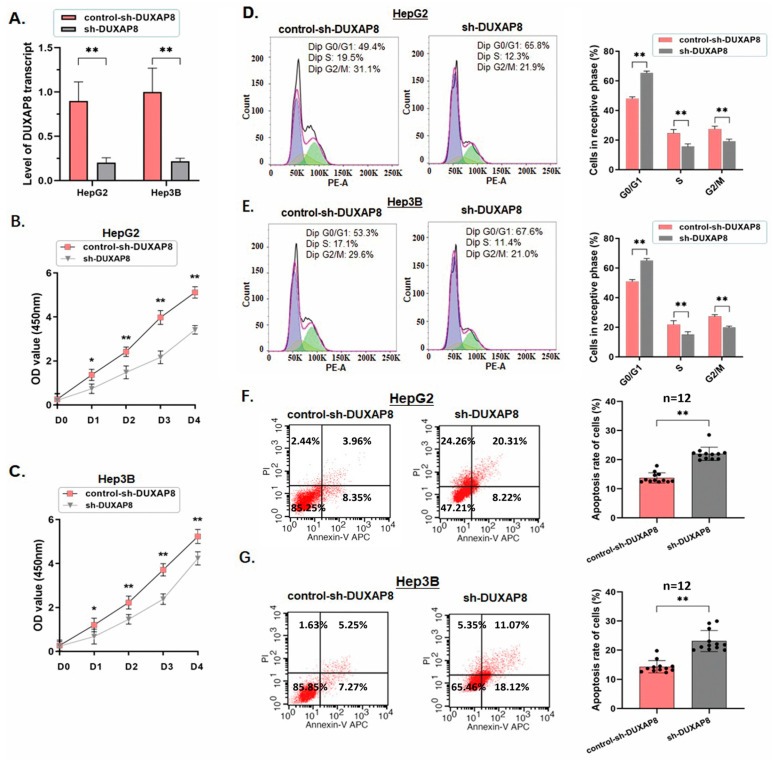
Knockdown of *DUXAP8* impaired HCC cell proliferation and induced cell apoptosis. (**A**) Knockdown of *DUXAP8* in HepG2 and Hep3B cells was conducted and validated by using the RT-qPCR assay (** *p* < 0.01). (**B**) The CCK8 assay was applied. Cell proliferation in HepG2 cells was significantly blocked after *DUXAP8* knockdown (* *p* < 0.05, ** *p* < 0.01). (**C**) Similar to the HepG2 cells, the cell proliferation of Hep3B cells was significantly blocked after *DUXAP8* knocking down (*p < 0.05, ** *p* < 0.01). (**D**) The representative histograms describing the cell cycle profiles of HepG2 cells by using flow cytometry are presented. For all histograms, the black and red lines indicate the raw data and the sum fit, respectively; the purple peak represents G0/G1 cells, while the green peak represents G2/M cells. The cell cycle of HepG2 cells was arrested in the G0/G1 phase by knockdown *DUXAP8*. The results are means of three independent experiments ± SD. (** *p* < 0.01). (**E**) The cell cycle of Hep3B cells was arrested in the G0/G1 phase after *DUXAP8* knockdown. The results are means of three independent experiments ± SD. (** *p* < 0.01). (**F**) Cell apoptosis rate was detected by using flow cytometry. The representative histograms show that the cell apoptosis rate of HCC cells was significantly increased from 13.25% to 25.54% for HepG2 cells. The results are means of three independent experiments ± SD. (** *p* < 0.01). (**G**) The cell apoptosis rate of HCC cells was significantly increased from 13.39% to 21.56% for Hep3B cells. The results are means of all independent experiments ± SD. (** *p* < 0.01).

HepG2 and Hep3B demonstrated the highest *DUXAP8* expression in our previous test and were selected for further tests. The *DUXAP8* transcript was knocked down using the shRNA tools in both cell lines, and was validated through an RT-qPCR assay (Figure 2A, Table S3). The following in vitro experiments indicated that the cell proliferation ability in HCC cells was remarkably repressed in those two cell lines when the *DUXAP8* transcript was knocked down (* *p* < 0.05; ** *p* < 0.01) (Figure 2B,C). A significant cell cycle arrest at the G0/G1 phases in the HCC cells was detected by the flow cytometric analysis in the cells with the *DUXAP8* transcript knocking down (Figure 2D,E). The percentage of the HepG2 and Hep3B cells in the G0/G1 phase increased, respectively, from 49.1% to 63.8% (*p* < 0.01) and from 50.2% to 64.2% (*p* < 0.01). The percentage of the cells in the S phase declined (HepG2: from 25.9% to 17.0%, *p* < 0.05; Hep3B: from 25.1% to 19.2%, *p* < 0.01), and, also, the G2/M phase (HepG2: from 23.9% to 16.7%, *p* < 0.01; Hep3B: from 25.8% to 19.1%, *p* < 0.01).

The calculated apoptotic HCC cells were increased by the flow cytometric analysis in both HepG2 and Hep3B cells (HepG2: from 13.25% to 25.54%, *p* < 0.01; Hep3B: from 13.39% to 21.56%, *p* < 0.01), sequentially induced by knocking down the *DUXAP8* transcript with significance. The above findings illustrated that the *DUXAP8* transcript exerts a potential promoting function by influencing cell growth and maintenance in HCC (Figure 2F,G).

### 2.4. Knockdown of DUXAP8 in HCC Cells Suppresses Tumor Growth and Lung Metastasis in the Orthotopic Transplantation Mouse Model

**Figure 3:**
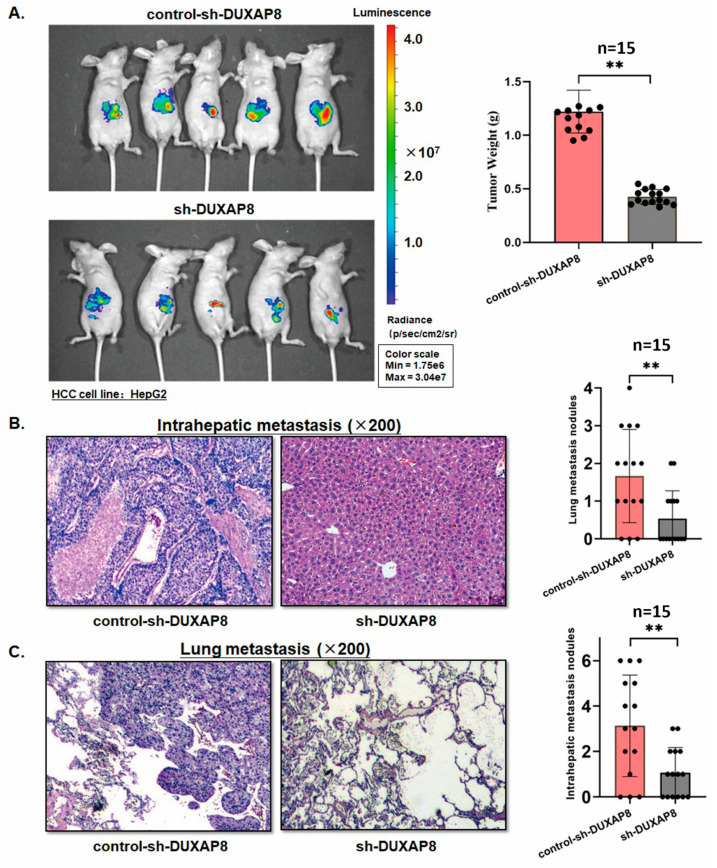
Knockdown of *DUXAP8* suppresses tumor formation and lung metastasis in vivo. (**A**) Mouse liver orthotopic transplantation model was constructed. The tumor growth at week 6 was significantly suppressed in the mouse models by *DUXAP8* knockdown in HepG2 cells. *DUXAP8* knockdown induced a significant decrease in the xenograft tumor weight in vivo (** *p* < 0.01). (**B**) The xenograft tumor specimens were collected at week 6 and examined under HE staining examination. Knocking down of *DUXAP8* led to fewer intrahepatic metastasis lesions in the mouse models than in the control ones (** *p* < 0.01). (**C**) Knocking down of *DUXAP8* resulted in fewer metastasis lesions in the lungs of the mouse models than in the control ones (** *p* < 0.01).

Xenograft mouse models were established by injecting the treated HepG2 cells. The orthotopically transplanted mass was quantified in livers six weeks post-orthotopic transplantation. The knocking down of *DUXAP8* in HepG2 cells led to significantly smaller tumor masses in mouse livers compared with the control ones (Figure 3A). Additionally, the graphics from the HE staining examination demonstrated that either intrahepatic metastasis or lung metastasis lesions were generated less in the mice models with *DUXAP8* knocking down than those in the control ones (Figure 3B,C).

The in vivo findings above support and complement our in vitro observation, and indicate that pseudogene *DUXAP8* transcription exerts important effects on tumor development and invasiveness. This pseudogene is a potential target for intensive investigation.

### 2.5. DUXAP8 Transcript Sponges miR-214-3p in HCC Cells

**Figure 4:**
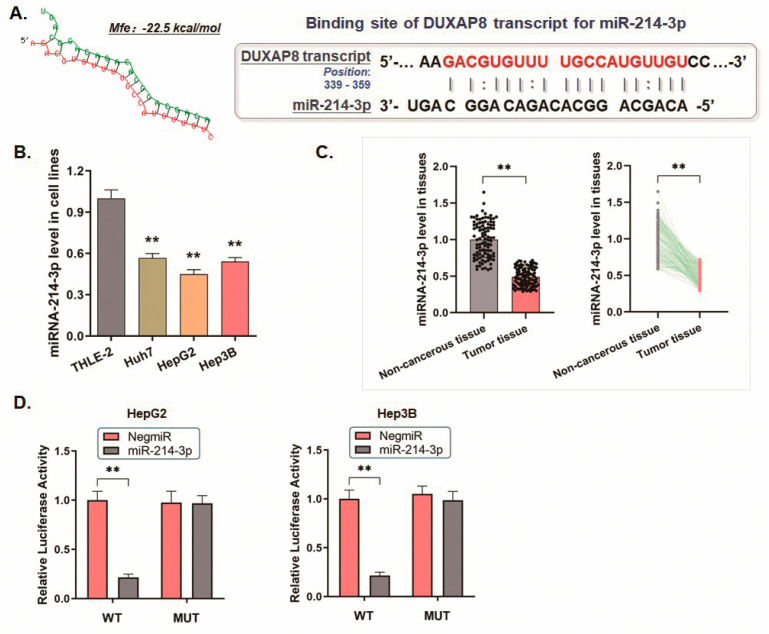
*DUXAP8* transcript sponges *miR-214-3p* in HCC cells. (**A**) The online microcosm software predicted that *miR-214-3p* could bind to the 3′-untranslated region (3′-UTR) of the *DUXAP8* transcript (the minimum free energy, Mfe: −22.5 kcal/mol). (**B**) The RT-qPCR assay indicated a significant decrease of *miR-214-3p* in HCC cell lines (** *p* < 0.01). (**C**) The expression of *miR-214-3p* was significantly downregulated in HCC tissues compared with the non-cancerous tissues (** *p* < 0.01). (**D**) The dual-luciferase reporter assay demonstrated that the signal suppressive effect induced by *miR-214-3p* was significantly impaired in the HCC cells transfected with a mutated binding site of *DUXAP8* transcript (** *p* < 0.01). This result indicates the sponging effect of the *DUXAP8* transcript on *miR-214-3p*.

By analyzing the sequence of the *DUXAP8* transcript and using the online microcosm prediction software, a short sequence from 339 bp to 359 bp to the 3′ end of *DUXAP8* transcript is predicted to match the seed region of *miR-214-3p* (the minimum free energy, Mfe: −22.5 kcal/mol), which prompts the use of the *DUXAP8* transcript as a probable competing endogenous RNA (ceRNA) targeting *miR-214-3p* (Figure 4A). Combined with the exploration of the HCC cell lines, we noticed that *miR-214-3p*, a reported inhibitor of HCC, was significantly downregulated in HCC (Figure 4B,C).

Based on the prediction, we constructed a mutated binding site in the *DUXAP8* transcript for the dual-luciferase reporter assay to validate the potential interaction between the *DUXAP8* transcript and *miR-214-3p*. The luciferase signal in either HepG2 or Hep3B cells transfected with *miR-214-3p* mimics was significantly decreased, based on the *DUXAP8*/pMIR/WT vector transfection, in comparison with the control ones. Conversely, the *DUXAP8*/pMIR/MUT vector transfection induced no significant signal change (Figure 4D, Table S4). All these findings above are consistent with the proposed ceRNA mechanism of the *DUXAP8* transcript in sponging *miR-214-3p*.

### 2.6. DUXAP8 Engages a Feedback Loop via Transcriptional Activation by miR-214-3p Targeted KLF13

**Figure 5:**
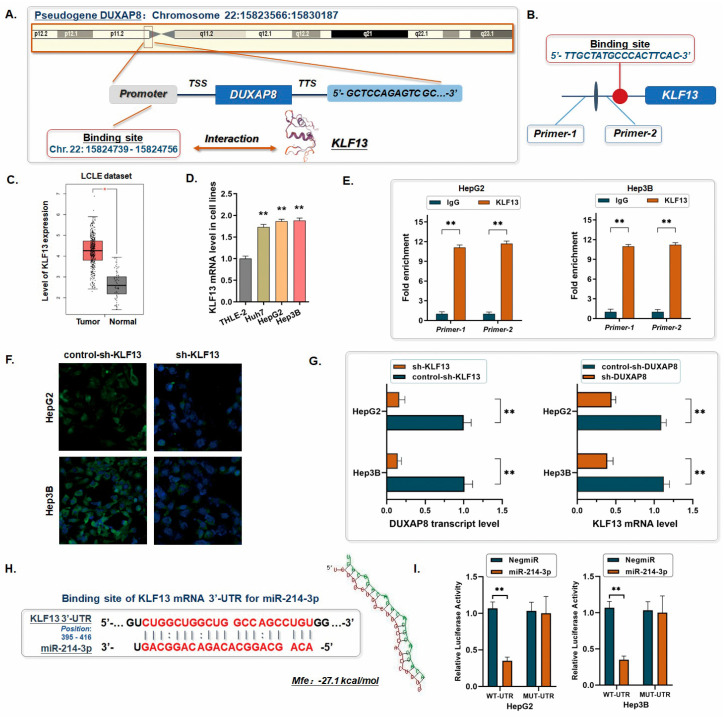
The feedback loop of *DUXAP8*/*miR-214-3p*/*KLF13*. (**A**) The 3000 bp fragment upstream of the pseudogene *DUXAP8*, which was regarded as its promoter region, was analyzed using the Database of Human Transcription Factor Targets. KLF13 was predicted as a probable transcription factor binding to the upstream sequence of pseudogene *DUXAP8*. (**B**) The binding site of the upstreaming region of *DUXAP8* (5 TTGCTATGCCCACTTCAC-3′) is presented in the histogram, and two primers (Primer 1 and Primer 2) were synthesized. (**C**) The expression of *KLF13* is significantly upregulated in HCC according to the TCGA datasets (* *p* < 0.05). (**D**) The level of *KLF13* mRNA in the HCC cell lines was potently upregulated compared with the THLE-2 cells (** *p* < 0.01). (**E**) The ChIP assay was carried out to investigate the direct interaction between KLF13 and the equivalent region of the promoter upstreaming pseudogene *DUXAP8* (** *p* < 0.01). IgG was used as the negative control. (**F**) Lentiviral vectors containing shRNA were used to deplete *KLF13* in Hep3G and Hep3B cells. The effect was validated by the immunofluorescence detection at 40× magnification, as shown. (**G**) Either knocking down *DUXAP8* or *KLF13* in the HCC cells sequentially induced a significant decrease in the expression of the other side (** *p* < 0.01). (**H**) *miR-214-3p* was predicted to bind to the 3′-untranslated region (3′-UTR) of *KLF13* mRNA (the minimum free energy, Mfe: −27.1 kcal/mol). (**I**) The dual-luciferase reporter assay was conducted. The signal suppressive effect induced by *miR-214-3p* was significantly impaired in the HCC cells transfected with the mutated binding site of the 3′-UTR of *KLF13* mRNA (** *p* < 0.01).

A 3000 bp fragment upstream of the first ATG of the pseudogene *DUXAP8*, regarded as the promoter region of *DUXAP8*, was intercepted for predicting the binding site of the potential transcription factor in tumorigenesis. Intriguingly, by evaluating through the Database of Human Transcription Factor Targets (https://guolab.wchscu.cn/hTFtarget/#!/, accessed on 13 October 2025), KLF13 was noticed as the probable transcription factor binding to the related region of the *DUXAP8* gene (5′-TTGCTATGCCCACTTCAC-3′, Chr. 22: 15,824,739-15,824,756) (Figure 5A,B). Accordingly, *KLF13* was highly expressed in HCC according to the TCGA liver cancer database and the HCC cell line exploration (Figure 5C,D). Thus, we conducted the ChIP assay to interrogate the interaction between these two genes and finally verified the predicted specific binding site for KLF13 on the promoter region of *DUXAP8* (Figure 5E, Table S5).

Notably, we observed similar trends of expression changes for either the *DUXAP8* transcript or *KLF13* mRNA by selectively knocking down the expression of the other side through in the HCC cells, and the knocking down of *KLF13* induced similar phenotypes in vitro to the knocking down of *DUXAP8* (Figure 5F,G, Figures S1 and S2). A post-transcriptional regulation between *miR-214-3p* and *KLF13* mRNA in the typical way was indicated. In brief, we used the Microcosm online software (https://www.microcosm.com/) and targeted *miR-214-3p* as an upstream regulator of *KLF13*, by potentially binding to the 3′-UTR of *KLF13* mRNA (Figure 5H). Then, the vectors containing the fragment of 3′-UTR from *KLF13* mRNA (WT-UTR) and the control luciferase vectors containing the mutated sequence (MUT-UTR) were constructed for the dual-luciferase reporter assay. The ectopic expression of *miR-214-3p* in HCC cells (HepG2/miR-214 and Hep3B/miR-214) significantly decreased the luciferase signal of *KLF13*/pMIR/WT, compared with the negative control (HepG2/NigmiR and Hep3B/NigmiR) (Figure 5I). The signal suppression induced by *miR-214-3p* was defective in the HCC cells transfected with a mutated binding sequence. The results above indicated a possible direct binding between *miR-214-3p* and *KLF13* mRNA, which suggested a feedback loop of *DUXAP8*/*miR-214-3p*/*KLF13*.

### 2.7. Feedback Loop of DUXAP8/miR-214-3p/KLF13 Impacts the Transcription of Piezo1

**Figure 6:**
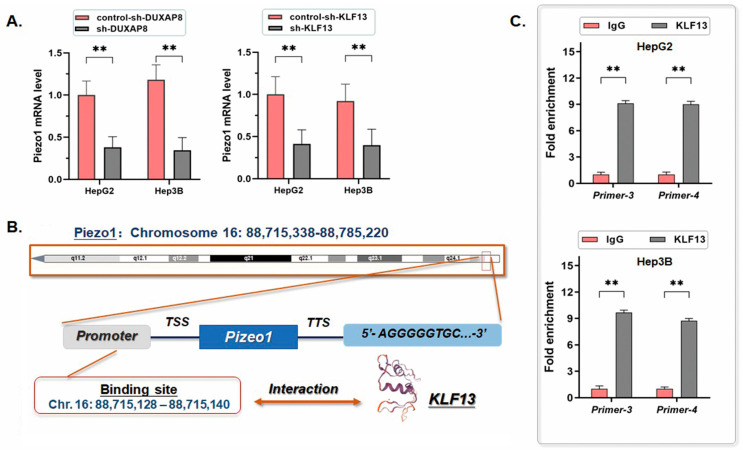
Feedback loop of *DUXAP8*/*miR-214-3p*/*KLF13* impacts the transcription of *Piezo1*. (**A**) The mRNA level of *Piezo1* in HCC cell lines was significantly decreased by knocking down either *DUXAP8* or *KLF13* (** *p* < 0.01). (**B**) The promoter region of *Piezo1* predicted as a potential binding site for KLF13 (5′-CTGCGGGAGGGGA-3′). (**C**) Two primers (Primer 3 and Primer 4) were synthesized for the ChIP assay. As shown in the ChIP assay histograms, the equivalent region of the promoter upstream *Piezo1* gene was directly bound by KLF13. IgG was used as the negative control (** *p* < 0.01).

*Piezo1* is a sensitive indicator of liver cancer, whose expression level changes along with the TME status and is commonly upregulated in HCC. In this study, we observed that knocking down either *DUXAP8* or *KLF13* in HCC cells induced the consequential decrease in *Piezo1* mRNA levels (Figure 6A, Table S6). Here, we detected the promoter region of *Piezo1*, and predicted a potential binding site for KLF13 (5′-CTGCGGGAGGGGA-3′, Chr. 16: 88,715,128–88,715,140). According to the ChIP assay results, we further validated the direct interaction between the transcription factor KLF13 and the specific sequence included in the *Piezo1* promoter region (Figure 6B,C, Table S7).

## 3. Discussion

As one of the major worldwide health challenges, HCC presents aggressive biological characteristics, leading to strong tumor growth, invasiveness, and motility phenotypes of the tumor cells [8]. The current progress in the treatment has promoted the outcomes of HCC patients to a certain extent, but rapid tumor progression and a high ratio of tumor recurrence after surgery largely limit the improvement of the OS and RFS. Hence, we are eager to understand more intensive intracellular mechanisms facilitating HCC aggression to explore better approaches to prevent and control this tumorous problem.

The current evidence has revealed the important roles of pseudogenes in human malignancies [9]. As described, the pathological transcript products of pseudogenes perform various functions throughout the whole process of tumor development, either promoting or suppressing tumorigenesis and progression [10]. The ceRNA effect is the most acknowledged function among the aberrant transcripts of pseudogenes verified in different human cancers by sponging miRNAs with a high affinity [11,12]. However, the complex functions of pseudogenes in HCC remain ambiguous. On the one hand, *INTS6P1* (a pseudogene of *Integrator complex subunit 6*, *INTS6*) is activated to transcribe and inhibits HCC cell proliferation by sponging tumor-related *miR-17-5p* [13]. On the contrary, *PCNAP1*, the pseudogene of *Proliferating Cell Nuclear Antigen* (*PCNA*), plays an opposite role in promoting HCC initiation in HBV-infected patients, which is induced by suppressing *miR-154* via a similar ceRNA mode [14].

Our recent research has revealed several signature pseudogenes that participate in HCC initiation and process through the application of the LCLE tools aforementioned. Innovatively, *AKR1B10P1*, *UBE2MP1*, and *SNRPFP1* were reported to be anomalously transcribed and function as a set of lncRNAs promoting HCC progression either in tumor growth or metastasis via individual mechanisms [15,16,17]. As noticed, these pseudogene transcripts might compose a cross-talk network to exert the ceRNA effects on different microRNAs and stabilize a series of coding RNAs impacting the HCC development. For further intensive study, we screened out *DUXAP8* as the co-expression pseudogene transcript for interrogation.

*DUXAP8* is a pseudogene of the *Double Homeobox Protein family member DUXA*, whose translational products are involved in early embryonic development [18]. The current literature has introduced several *DUXA*-derived pseudogenes, like *DUXAP9* and *DUXAP10*, which were sporadically reported as oncogenes in several human malignancies, including osteosarcoma, leukemia, and non-small-cell lung cancer [19,20,21]. As for the pseudogene *DUXAP8*, its length is 2307 bp, and the transcript is regarded as an lncRNA, which has a nucleotide composition over 200nt [22]. However, the exact bio-information of the parental gene *DUXA* is quite limited and provides no valuable details about its daughter pseudogene *DUXAP8*.

Currently, the abnormal overexpression of the *DUXAP8* has been discovered in some human cancers (e.g., pancreatic cancer, lung adenocarcinoma, and gastric cancer), potentially associated with oncogenic events in most of them by affecting the proliferation, motility, and anti-autophagy of tumor cells [23,24,25]. The expression of *DUXAP8* in HCC has been noticed and is generally thought to promote HCC by different bio-processes, including sponging microRNAs (*miR-490* and *miR-422*) and enhancing cell proliferation [26,27]. According to our observations comparing HCC tumor tissues with paracancerous liver tissues, *DUXAP8* was potently activated in tumors, along with the detectable transcript in tumor cells. A more highly expressed *DUXAP8* was correlated with dismal clinicopathologic features, such as a higher AFP level and advanced tumor stage characteristics (microsatellite formation, venous invasion, and liver cirrhosis). Simultaneously, the loss of function of *DUXAP8* in our study demonstrated that knocking down *DUXAP8* in HCC cells remarkably impairs the cell proliferation ability and facilitates the cell apoptosis process. We hypothesize that *DUXAP8* overexpression contributes to HCC progression. According to the in vivo examination, the HCC tumor formation treated by *DUXAP8* knockdown was significantly suppressed in both the liver orthotopic transplantation and the lung metastasis lesions. These findings further elucidate *DUXAP8* as a promising HCC oncogene.

The exploration in our study on the potential ceRNA role of *DUXAP8* screened *miR-214-3p* as one of the targets, which shares the binding sites directly matched with the specific sequence of the *DUXAP8* transcript. In the literature, *miR-214-3p* presents an aberrant profile of the expression in multiple malignancies, including breast cancer, colorectal cancer, and liver cancer, and mostly plays the role of a tumor suppressor [28,29,30]. For HCC, *miR-214-3p* is commonly downregulated in tumor tissues, and its ectopic expression through mimics induced the obvious inhibition of either cell proliferation or migration by degrading the mRNAs involved in the MAPK1 or HOTAIR signaling pathway [31,32]. Herein, we validated the direct interaction between the *DUXAP8* transcript and *miR-214-3p* and observed a significant downregulating effect of *DUXAP8* knockdown on the degradation of *miR-214-3p*.

Sequentially, we interrogated *miR-214-3p* by the luciferase reporter assay and verified it as an upstream regulator of KLF13, which is the transcription factor involved in various cancers. As acknowledged, *KLF13* is a pivotal member of the Krüppel-like factor (KLF) family, containing conserved zinc-ester domains for regulating transcriptional activity. *KLF13* has been reported to be highly expressed in oral cancer cells and to significantly promote cell proliferation [33]. Recently, researchers have described *KLF13* as a overexpressed promoter in HCC, which mediates and enhances HMGCS1-related cholesterol biosynthesis [34]. However, the exact mechanism underlying *KLF13* expression in HCC remains unclear. In this study, our findings demonstrated that the knocking down of *DUXAP8* in the HCC cell lines induced a significant decrease in *KLF13*. Thus, we hypothesize that the abnormal transcription of *DUXAP8* could exert the function of stabilization and a high expression status of *KLF13* via the molecular sponging effect.

Importantly, we evaluated the probable downstream effectors of KLF13 in HCC to better understand the underlying mechanism. Interestingly, the pseudogene *DUXAP8* was occasionally predicted as one of the *KLF13* targets, sharing the potential binding site upstream of the *DUXAP8* sequence. The ChIP assay showed that KLF13 directly binds to the promoter region of *DUXAP8*. Hence, we suggested that *DUXAP8* and *KLF13* consist of a positive feedback loop through the ceRNA effect on *miR-214-3p*, which affects the HCC process, probably in an amplification mode in the TME.

Does this feedback loop promote HCC progress by modulating some critical effectors? Based on this point of view, the TME-related *Piezo1* was selected as another predicted candidate downstream of KLF13, and this gene may be a pivotal effector affected by the *DUXAP8*/*miR-214-3p*/*KLF13* feedback loop. In brief, *Piezo1* is widely distributed in various human organs and tissues, like the cardiovascular, lung, urinary, and immune systems [35]. Structurally, Piezo1 plays a specific role in mechanosensitive ion channels and functions in the activation of cell signaling pathways concerning mechanical stimulation [36].

Mechanical stress caused by tissue stiffening is the predominant characteristic in the composition of the liver TME [37]. Liver fibrosis and related cirrhosis are the most important causes of HCC tumorigenesis based on different inducements, like acute or chronic liver injury, and persistent hepatitis. Moreover, the TME of HCC involves complex intrinsic and extrinsic factors with a high heterogeneity that contribute to liver fibrosis, sclerosis, and permeability transition. Therefore, we believe that the expression change in *Piezo1* is an innovative indicator of the liver TME status in the context of liver inflammation, fibrosis, or cirrhosis. Similarly, accumulating evidence has demonstrated that *Piezo1* was upregulated in the hepatic fibrosis process and contributed to HCC progression. Although we did not further investigate the downstream effectors of Piezo1, the literature suggests that its activation can induce a range of cellular changes, including hypoxia tolerance, the inhibition of apoptosis, and the epithelial–mesenchymal transition. One piece of convincing evidence is that highly expressed Piezo1 activates the MAPK pathway in the YAP cascade by inducing the phosphorylation of JNK, p38, and ERK, and promotes HCC growth in vivo [38].

In our study, the modulation of the *DUXAP8*/*miR-214-3p*/*KLF13* feedback loop could significantly affect *Piezo1* transcription and HCC growth. This finding is consistent with the positive role of *Piezo1* in HCC progression. We propose that *DUXAP8*, along with the members of this feedback loop, participate in the regulation of *Piezo1* as a series of practicable indicators for liver TME and HCC progression. However, it is worth noticing that, though our results strongly indicate ceRNA regulation in the feedback loop, we cannot fully rule out parallel regulatory mechanisms with the current evidence. Further study would be needed to explore other possible pathways in this loop.

Last but not least, in the following research, we will focus on the intensive modulation mechanism induced by *DUXAP8* via Piezo1, which has to be interrogated intensively, and the exact effects of *Piezo1* on HCC TME will be further detected under the background of the *DUXAP8*/*miR-214-3p*/*KLF13* feedback loop as well.

In summary, this study demonstrates a featured feedback loop in HCC progress predominantly triggered by the abnormally transcribed pseudogene *DUXAP8*, and the members of the loop not only stabilized the expression of *DUXAP8* through a ceRNA way but also positively modulated the expression of the *Piezo1*. Our work probably provides a set of innovative indicators and therapeutic targets in HCC prevention.

## 4. Materials and Methods

### 4.1. Cell Culture and Preparation

Three typical HCC cell lines (Huh7, HepG2, and Hep3B) were cultured along with the control transformed human liver epithelial-2 (THLE-2) (Shanghai Institutes for Biological Sciences, Chinese Academy of Science, Shanghai, China). Briefly, the cell lines were respectively cultured in RPMI 1640, supplemented with 10% heat-inactivated fetal bovine serum (FBS), incubated at 37 °C, with 100 μg/mL streptomycin, and 100 U/mL Penicillin in a humidified atmosphere of 5% CO_2_. Cells were authenticated by short tandem repeat (STR) profiling and routinely tested for mycoplasma contamination using PCR and colorimetric assays, and only mycoplasma-free cells were used for experiments. Specifically, for the transfected cells, a medium containing G418 (Santa Cruz Biotechnology, Inc., Dallas, TX, USA; 400 μg/mL) was used for selection. The selection process was maintained until non-transfected control cells were completely eliminated, lasting for 5–7 days.

### 4.2. Clinical Specimens

Ninety-five pairs of HCC-related specimens containing the tumor tissues and the paracancerous liver tissues at a 1 cm distance from the tumor margin were collected. All patients underwent R0 radical resection without any pre- or post-operative treatment at the Department of General Surgery, Ruijin Hospital, Shanghai Jiao Tong University School of Medicine (2016 to 2020). The clinicopathologic features of those patients were organized, including gender, age, tumor size, number of lesions, grades, et al. The study was approved by the Ethics Committee of Ruijin Hospital, Shanghai Jiaotong University School of Medicine (No. 2021-421), and informed consent was obtained.

### 4.3. Preparation and Application of the Datasets

The differential gene expression for 369 liver tumors and 50 normal samples from the UCSC Xena database, combined with an additional 110 normal liver samples data from the GTEx and TCGA, were intensively explored by using the random-walk-based multi-graphic (RWMG) model algorithm developed by our team [39]. The relative information of the pseudogene-derived transcripts was analyzed and presented in the aforementioned LCLE tools. The starBase datasets (https://starbase.sysu.edu.cn/, accessed on 13 October 2025) and the dreamBase (https://ngdc.cncb.ac.cn/databasecommons/database/id/3746, accessed on 13 October 2025) datasets were introduced to provide much more supplementary information on the expression and relationship of the candidate genes.

### 4.4. RT-qPCR Assay and Immunohistochemistry Assay

RNA isolation from tissues or cells is performed according to the instructions of the TRIzol reagent (Invitrogen, Waltham, MA, USA). The first-strand cDNA was synthesized via a High-Capacity cDNA Reverse Transcription Kit (ABI, Los Angeles, CA, USA). All the primers were synthesized by Jike Biotech Company (Shanghai, China) (Table S1). Real-time quantitative polymerase chain reaction (RT-qPCR) was performed following the TaqMan Gene Expression Assays protocol (ABI, USA). The relative quantification of RNA in cell lines was normalized using GAPDH by the 2 − Δ*C^t^* method. The relative quantification of *miR-214-3p* in tissue specimens and cell lines was measured by using the mirVANATM miRNA Isolation Kit (ABI, USA). The PCR program was set as follows: 95 °C for 10 min, followed by 35 cycles of 95 °C for 15 s, 60 °C for 30 s, and 72 °C for 45 s.

Antibodies against KLF13 were purchased from Abcam, Waltham, MA, USA. The immunohistochemistry assay (IHC) complied with our previously described methods [40]. The protein expression levels detected by IHC were independently assigned to two experienced pathologists for blind examination and were separated into two groups based on staining intensity grade: no to low staining (0~1+) and moderate to high staining (2+~3+).

### 4.5. Plasmid Preparation and Cell Transfection

The lentiviral vectors pLKO.1 containing shRNA were transfected into cultured HepG2 and Hep3B cells at the exponential phase (JIKE Biochemistry, Shanghai, China) to suppress the expression of the *DUXAP8* transcript, and the control vectors were used. The transfected cells were selected using a medium containing G418 (Santa Cruz Biotechnology, Inc.; 400 μg/mL). The mimic was used to transfect HCC cells for ectopically introducing *miR-214-3p* (HepG2/miR-214; Hep3B/miR-214), along with a set of negative controls (HepG2/NigmiR; Hep3B/NigmiR). The lentiviral vector pLV (Addgene, Cambridge, MA, USA) was applied for ectopically expressing *KLF13* (pLV-KLF13) for the rescue experiments, and the pLV-Null was set for control.

### 4.6. Cell Proliferation and Cell Cycle Detection

The HCC cells (1 × 10^6^) were cultured in 96-well microtiter plates, triplicated, and incubated at an atmosphere of 5% CO_2_ and 37 °C for 5 days. Microplate computer software version 1.30 (Bio-Rad Laboratories, Inc., Hercules, CA, USA) was used to measure the OD following the Cell Counting Kit-8 (CCK-8) assay kit protocol (Dojindo, Tokyo, Japan), and the cell proliferation curves were plotted. The cells were fixed with ethanol, and then treated with RNase A and stained using propidium iodide. Flow cytometry detection was carried out using FACSCalibur (Becton-Dickinson, Franklin Lakes, NJ, USA), and ModFit software version 5.0 (Becton–Dickinson, Franklin Lakes, NJ, USA) was used for quantifying cell populations at the G0/G1, S, and G2/M phases (the debris and fixation artifacts of the cells were excluded).

### 4.7. Cell Apoptosis Analysis

Cell apoptosis rate was calculated using PE-Annexin V Apoptosis Detection Kit I (BD Pharmingen, Franklin Lakes, NJ, USA) according to the instructions. Transfected cells were resuspended in a concentration of 1 × 10^6^ cells/mL in the 1 × Binding Buffer. Then, 5 μL of FITC and 5 μL of PI were added into 100 μL of the cell suspension, followed by a 15-min incubation in darkness, with 400 μL × Binding Buffer added. The apoptosis rate was determined by flow cytometry (Becton Dickinson, Franklin Lakes, NJ, USA). FSC/SSC gating was used to exclude debris and FSC/SSC-B gating was used to exclude doublets. Cells were classified as follows: Annexin V^−^/PI^−^ viable cells, Annexin V^+^/PI^−^ early apoptotic cells, and Annexin V^+^/PI^+^ late apoptotic cells. Both early and late apoptotic cells were used to calculate the apoptosis rate. Appropriate unstained controls and single-stained controls were used for compensation.

### 4.8. The Mouse Liver Orthotopic Transplantation and Tumorigenicity Assay

The function of *DUXAP8* in HCC development was assessed by the tumorigenicity assay. Five-week-old male BALB/c nude mice (Institute of Zoology, Chinese Academy of Sciences, Beijing, China) were purchased and housed in a pathogen-free environment and were randomized into the treatment and control groups (fifteen mice for each group). All protocols were performed in accordance with the guidelines of the Shanghai Medical Experimental Animal Care Commission, including the randomization and blinding statement. A total of 1 × 10^6^ HCC cells were suspended within the 25 µL serum-free DMEM, which was mixed with 25 µL Matrigel (1:1, *v*/*v*). Cells were orthotopically injected into the left hepatic lobes of the mice. The mice were monitored regularly for general condition and sacrificed at 6 weeks after injection. Investigators were blinded to group allocation during outcome assessment. The weight of all the xenografted livers was measured, and the mice’s livers and lungs were stained with hematoxylin and eosin (HE) for pathological examination. All animal experiments were approved by the IACUC of Ruijin Hospital, Shanghai Jiao Tong University School of Medicine (No. 2020-148), and were performed in accordance with relevant guidelines and regulations.

### 4.9. Dual-Luciferase Reporter Assay

*MiR-214-3p* was predicted to be a potential regulator of *KLF13* using the Microcosm online tool (https://www.targetscan.org/vert_80/). We selected a 202 bp sequence containing the putative *miR-214-3p* binding site at the 3′-UTR of *KLF13* mRNA and designed the mutant sequence (Table S2). The sequences were respectively cloned into the pMIR-Report luciferase vector, which contains firefly luciferase, and the pRL-TK vector luciferase was used as a control (Promega, Madison, WI, USA). These two sets of vectors were co-transfected into the HCC cells, introducing *miR-214-3p* or the controls. The luciferase activity was measured via the Dual-Glo Luciferase assay system (Promega) 48 h after the transfection. Simultaneously, we constructed the sequence and the relative mutated sequences, containing the putative binding site of *miR-214-3p* of *DUXAP8* transcripts (Table S2).

### 4.10. Chromatin Immunoprecipitation Assay

The chromatin immunoprecipitation (ChIP) assay was performed to verify the interaction between the transcription factor and the promoter region of targeted genes (KL13 to *DUXAP8* or *Piezo1*). A total of 5 × 10^6^ cells were cultured in each 10 cm dish and subjected to ChIP assay following ChIP-ITTM Kit’s protocol (Active Motif, Carlsbad, CA, USA). Chromatin was immunoprecipitated with 2 μg of either a transcription factor antibody (Abcam, USA) or IgG as the negative control. The extracted DNA was analyzed through RT-qPCR by using the relative primers (Table S1).

### 4.11. Statistical Analysis

Statistical analysis was conducted by using SPSS 20.0. Each experiment was repeated three times independently, and the statistical analysis was conducted according to the mean of these results. *p*-values were calculated following an unpaired Student’s *t*-test and Fisher’s exact test. Spearman and Logistic analysis were used to describe the association between the various parameters and the risk of *DUXAP8* status. Differences were considered statistically significant at *p*-values < 0.05 in this study.

## Figures and Tables

**Table 1:** Correlation between *DUXAP8* transcript and the clinicopathological features in 95 HCC specimens. The level of *DUXAP8* transcript associated with clinicopathologic features in 95 HCC patients, including age, gender, tumor size, tumor stage (AJCC), tumor encapsulation, tumor microsatellite formation, vein invasion, HBsAg status, AFP level, and liver cirrhosis. Statistically, significance was assessed by Fisher’s exact test.

Clinicopathologic Parameters	DUXAP8		*p* *
	High (*n* = 89)	Low (*n* = 6)	
**Age (years)** ≤50 >50			
	56	3	0.670
	33	3	
**Gender** Male Female			
	47	4	0.683
	42	2	
**Diameter (cm)** ≤5 >5			
	41	5	0.104
	48	1	
**TNM stage** I~II III~IV			
	22	4	0.046^*^
	67	2	
**Tumor encapsulation** Absent Present			
	33	3	0.670
	56	3	
**Tumor microsatellite formation** Absent Present			
	32	5	0.032 *
	57	1	
**Venous invasion** No Yes			
	25	5	0.011 *
	64	1	
**HBsAg** Negative Positive			
	9	2	0.142
	80	4	
**AFP(ng/mL)** ≤400 >400			
	15	5	<0.001 *
	74	1	
**Cirrhosis** Absent Present			
	8	2	0.119
	81	4	

*p* * < 0.05.

## Data Availability

Data from this study is not made public due to privacy regulations but is available upon request from the authors via sending a message to Junqing Wang (e-mail: wangjunqingmd@hotmail.com).

## Supplementary Materials

The following supporting information can be downloaded at: https://www.mdpi.com/article/10.3390/ijms27114873/s1.

## Abbreviations

Hepatocellular carcinoma (HCC); overall survival (OS); tumor microenvironment (TME); extracellular matrix (ECM); Piezo-type mechanosensitive ion channel component 1 (*Piezo1*); KLF Transcription Factor 13 (*KLF13*); the chromatin immunoprecipitation assay (ChIP assay); 3′-untranslated region (3′-UTR); and competing endogenous RNA (ceRNA).
