# Supplementary material for: Feedback Loop of DUXAP8/miR-214-3p/KLF13 Facilitates Hepatocellular Carcinoma Progression and Serves as an Indicator of Tumor Microenvironment via Impacting Piezo1
Source: Int J Mol Sci. 2026 May 28;27(11):4873. doi: 10.3390/ijms27114873 (PMC13256093; doi:10.3390/ijms27114873)
Supplement: Supplementary file 1 [file ijms-27-04873-s001.zip › ijms-4269932-supplementary.pdf]

Suppl. Table. S1

The primers for RT-qPCR assay or ChIP assay

Genes	Forward	Reverse
DUXAP8	5’- CCCCTCTTCACACAAGATGGT-3’	5’- GGGGAGAAGTGTGTTCTACC-3’
KLF13	5’- TCACAGACCCACGGTTTTCC -3’	5’- TGGAGACACATCCTTTGCCC -3’
miR-214-3p	5’-<br>ACACTCCAGCTGGGACAGCAGGCAC<br>AGACA-3’	5’-<br>CTCAACTGGTGTCGTGGAGTCGGC<br>AATTCAGTTGAGACTGCCTG-3’
Pizeo1	5’-TCCTCAACCACATGGTCACG-3’	5’-GCGATCTCGGTGAAGACGAT-3’
Primer 1	5’-TGCCCGTAAGAGACATGTGG-3’	5’-TCAGCCTCCCAAAGTGCTG-3’
Primer 2	5’-TGTGAGACTATGCAAATGCTGA-3’	5’-ATTACAAGTGCCTGCCACCA-3’
Primer 3	5’-GACCCCTTATGTAGCGCCC-3’	5’-CAGTCGGGGGTCTCCAGG-3’
Primer 4	5’-CTCGGGGAGCAAAGCGAAAG-3’	5’-CCTTTATAAGCTCGGCCCCC-3’

Suppl. Table. S2

The selected sequence of the predicted miR-214-3p binding site of the 3’-UTR of KLF13 mRNA and the DUXAP8 transcript, along with the relative mutated sequences

Genes	Sequence including the binding site (202 bp)	Corresponding mutated sequence
KLF13 mRNA 3’-UTR	5’-<br>ctgtacatagatttgcactctggagttttctgttaggttcggg<br>aacacgctggggacagagcaggccagccagtctggtgg<br>agctcagcgtctggctggctggccagcctgtgggtctgtt<br>gggagcagatgtgctcactgacgtttgtgcctccagggca<br>gattcctaagcagctggcctgacctcttcctgcccagcccc<br>-3’	5’-<br>tcttctttgttatcctcacagcactatacagattgctaccgcat<br>ctcccagcgcagacacctgccgaccgactgtcgagcacc<br>actgggacagccagccagccgaccgtctcgctgtctagcg<br>tggacaagaggtgagtcaggatagaggcacgacgcctgtt<br>acgttacctggtcggcagtcgtgtacgtccgctggcgc-3’
DUXAP8 transcript	5’-<br>tttgttttaaataattgagcttcaagtaagggcagcaagtagt<br>ttttcttctttttctttctgtttttctttttcttttcttttttttaaagacg<br>tgttttgccatgttgtccaggctggtcttgaactcctgaattc<br>aagtaatcaacctgtcttggcctcccaaagtgatgggatta<br>caggtatgagccaccgaacccag-3’	5’-<br>atagatattatttaatcaccatgatgaatgcggacctacttgat<br>atacatgtatatgtatgtctatatgtatatacatatgtatatatta<br>tgtcctctataggcttctagacgacggtcgacatcatcacgt<br>cattactacttaactagcagacatcggcacgctatgagttcg<br>caattctgctttcaccgagccatcgctg-3’

Suppl. Table. S3

RT-qPCR results of DUXAP8 expression in HepG2 and Hep3B after knockdown of DUXAP8

Cell lines	control-sh-DUXAP8	sh-DUXAP8
**HepG2**	0.95±0.15	0.21±0.05
**Hep3B**	0.99±0.16	0.22±0.06

Suppl. Table. S4

The relative luciferase activity in both wildtype and muatated cell lines of HepG2 and Hep3B transfected with negative control miRNA or miR-214-3p

Cell lines	WT		MUT	
	NegmiR	miR-214-3p	NegmiR	miR-214-3p
**HepG2**	1.00±0.06	0.23±0.03	1.02±0.03	0.99±0.07
**Hep3B**	1.00±0.08	0.21±0.02	1.15±0.09	1.08±0.08

Suppl. Table. S5

The ChIP assay results demonstrates KLF13 binding at the DUXAP8 promoter in HepG2 and Hep3B cells

Cell lines	Primer-1		Primer-2	
	IgG	KLF13	IgG	KLF13
**HepG2**	0.93±0.37	11.00±0.42	0.92±0.33	11.60±0.21
**Hep3B**	0.93±0.49	10.88±0.33	0.94±0.44	11.14±0.35

Suppl. Table. S6

The mRNA level changes of Piezo1 in HepG2 and Hep3B after knocking down either DUXAP8 or KLF13

Cell lines	control-sh-DUXAP8	sh-DUXAP8	control-sh-KLF13	sh-KLF13
**HepG2**	1.00±0.13	0.42±0.04	1.00±0.11	0.37±0.04
**Hep3B**	1.09±0.06	0.43±0.06	1.00±0.1	0.34±0.03

Suppl. Table. S7

The ChIP assay results demonstrates KLF13 binding at the Piezo1 promoter in HepG2 and Hep3B cells

Cell lines	Primer-3		Primer-4	
	**IgG**	**KLF13**	**IgG**	**KLF13**
**HepG2**	0.92±0.45	9.21±0.45	0.92±0.41	8.73±0.28
**Hep3B**	0.96±0.34	9.62±0.51	1.03±0.34	9.52±0.41

Supplementary Figures

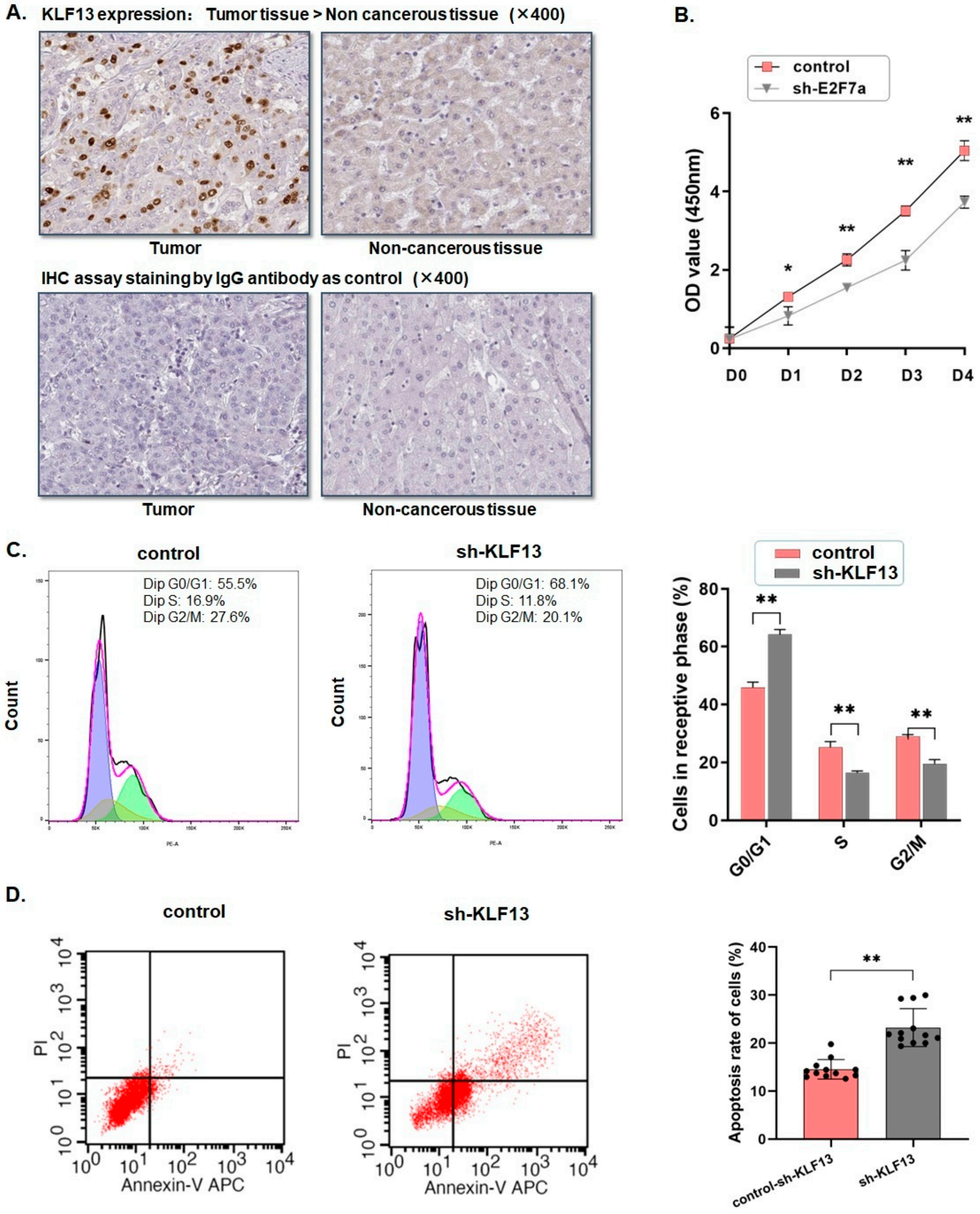

Suppl. Fig. S1. KLF13 expression profile in HCC and the in vitro experiment after depleting KLF13 in the cell line

A. The representative graphs of immunohistochemistry analysis (400×) of HCC patients' specimens. Specimens were regarded as the control by staining IgG antibodies. KLF13 expression in tumor tissues was significantly higher than in adjacent non-cancerous tissues. **B.** The CCK8 assay was applied. The HCC cell proliferation (HepG2) was significantly suppressed by depleting KLF13 (**P*< 0.05, ***P*< 0.01). **C.** Flow

cytometry was conducted for detecting the cell cycle. The representative dot plots show that the cell cycle of HCC cells was arrested in the G0/G1 phase by depleting KLF13. The results are means of three independent experiments ±SD. (***P*< 0.01). **D.** Cell apoptosis rate was detected by flow cytometry exploration. The representative histograms describe that the cell apoptosis rate of HCC cells was significantly increased in HepG2 cells after KLF13 depletion. The results are means of three independent experiments ±SD. (***P*< 0.01).

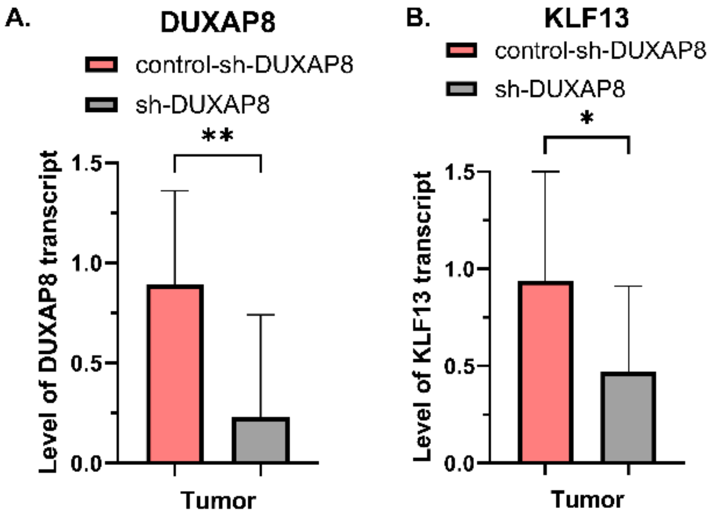

Suppl. Fig. S2. DUXAP8 and KLF13 expression level in xenograft tumor

A. RT-qPCR assay was performed to validate the knockdown of DUXAP8 in the xenograft tumor. (***P*<0.01). **B.** RT-qPCR assay demonstrates the decline of KLF13 after DUXAP8 depletion *in vivo* (**P*<0.05).
